# Polyvinyl Alcohol/Chitosan and Polyvinyl Alcohol/Ag@MOF Bilayer Hydrogel for Tissue Engineering Applications

**DOI:** 10.3390/polym13183151

**Published:** 2021-09-17

**Authors:** Meng Zhang, Guohui Wang, Xin Zhang, Yuqi Zheng, Shaoxiang Lee, Dong Wang, Yang Yang

**Affiliations:** 1 College of Environment and Safety Engineering, Qingdao University of Science and Technology, Qingdao 266042, China; A903962566@qust.edu.cn (M.Z.); 17864298538@qust.edu.cn (G.W.); 13176388366@qust.edu.cn (X.Z.); marcella_Zheng@qust.edu.cn (Y.Z.); 2 Shandong Engineering Research Center for Marine Environment Corrosion and Safety Protection, Qingdao University of Science and Technology, Qingdao 266042, China; 3 Shandong Engineering Technology Research Center for Advanced Coating, Qingdao University of Science and Technology, Qingdao 266042, China; 4 National Marine Data and Information Service, Tianjin 300171, China; yangyangouc@qust.edu.cn

**Keywords:** Ag-Metal-organic framework, polyvinyl alcohol, antibacterial activity, cytotoxicity

## Abstract

In this paper, polyvinyl alcohol/Ag-Metal-organic framework (PVA/Ag@MOF) and polyvinyl alcohol/chitosan (PVA/CS) were used as the inner and outer layers to successfully prepare a bilayer composite hydrogel for tissue engineering scaffold. The performance of bilayer hydrogels was evaluated. The outer layer (PVA/CS) has a uniform pore size distribution, good water retention, biocompatibility and cell adhesion ability. The inner layer (PVA/Ag@MOF) has good antibacterial activity and poor biocompatibility. PVA, PVA/0.1%Ag@MOF, PVA/0.5%Ag@MOF, and PVA/1.0%Ag@MOF show anti-microbial activity in ascending order. However, its use as an inner layer avoids direct contact with cells and prevents infection. The cell viability of all samples was above 90%, indicating that the bilayer hydrogel was non-toxic to A549 cells. The bilayer hydrogel scaffold combines the advantages of the inner and outer layers. In summary, this new bilayer composite is an ideal lung scaffold for tissue engineering.

## 1. Introduction

Tissue engineering scaffold are materials that mimic the extracellular matrix (ECM) environment to bind to living tissue cells and can be implanted into living organisms [1,2,3,4,5,6]. The ideal tissue engineering material should essentially have good biocompatibility and good mechanical properties [7,8]. For optimal tissue regeneration, the scaffold should be biodegradable and the rate of scaffold degradation should match the rate of new tissue formation [9]. However, scaffolds that degrade slowly are usually preferred in practice to minimize the risk associated with premature absorption of this scaffold.

Hydrogels are cross-linked three-dimensional polymer networks which can retain and absorb a great deal of water [10,11,12,13]. Both natural and synthetic polymers can be physically and chemically cross-linked to prepare hydrogels [14,15]. Due to their high water content, hydrogels are highly biocompatible, as well as having rubbery mechanical properties close to those of soft tissues, and often allow the incorporation of cells and bioactive molecules during the gelling process.

Chitosan is a polycationic natural polysaccharide biopolymer widely used in the manufacture of hydrogels [16]. Chitosan is a biocompatibility material with non-toxic and antibacterial activity [17]. The amino group on the chitosan interacts with the negative charge on the red blood cell membrane, which cause it to have an anticoagulation effect. Chitosan can promote the proliferation of fibroblasts, enhance the generation of the extracellular matrix and the activation of macrophages [18]. As chitosan hydrogels are prepared by chemical cross-linking, their biocompatibility is poor. Therefore, chitosan is blended with other polymers (polyvinyl alcohol) to prepare hydrogels with good biocompatibility by freeze–thaw cycling to expand its application in biomedical fields.

Polyvinyl alcohol as an extremely safe polymer binder, its hydrogel has a similar water content to natural human tissue, high mechanical strength, good biocompatibility, and porous network structure as well as is widely used in biomedicine [19,20]. There are certain complications in the transplantation process, such as malabsorption which can lead to a serious risk of infection. The presence of silver ions virtually eliminates the risk of bacterial infection [21].

Silver-based compounds and silver nanoparticles (AgNPs) have been shown to have strong antibacterial activities and, thus, act as alternatives to antibiotics [22,23,24]. Silver sulfadiazine and AgNPs, for example, have become topical antibacterial cream for burns and wounds [25,26]. Although AgNPs are famous for their high bactericidal effects connected with the release of Ag^+^, they tend to form aggregates when they come into contact with bacteria, which limits their activity due to loss of active surface area [27,28,29]. Thus, it is still essential to design new materials with controllable structures as efficient media to overcome the limitation.

Metal-organic frameworks (MOFs), a relatively new type of the inorganic-organic hybrid polymer, have exhibited their potential application for gas storage [30], gas separation [31], sensors, catalysis, and advanced biomedicine, as well as drug delivery [32,33]. However, the bactericidal applications of MOFs have rarely been explored up to now. Compared with traditional bactericide, MOF antibacterial agent has the advantages of broad antibacterial spectrum, high effectiveness, long-acting, tunable structures, and thermal stability [34]. Ag@MOFs, composed by Ag^+^/clusters and the organic ligands, have the potential application in antimicrobial materials against bacteria, yeast and mold [35]. The functional groups of the organic ligands can strongly interact with the polymer matrix, resulting in hydrogen bond, which improves the mechanical properties of films. Ag@MOFs with the special rod-like structures not only result in homogeneous dispersion of it in polymer matrix, but also sustained release Ag^+^ to avoid aggregation. In addition, Ag@MOFs have more excellent antibacterial activities compared with Ag-NPs, which were related to (i) the disruption of cells owing to the penetration of bacterial cells by MOFs, (ii) the bacterial membranes damage caused by the interaction of silver ions with thiol proteins, (iii) the combination between the bacterial cell cations including Mg^2+^ and the functional groups of the organic linkers, and (iv) the release of reactive oxygen species (ROS) [29]. However, the cytotoxicity of Ag@MOF is high, so we have designed a bilayer hydrogel to avoid this limitation.

**Figure 1:**
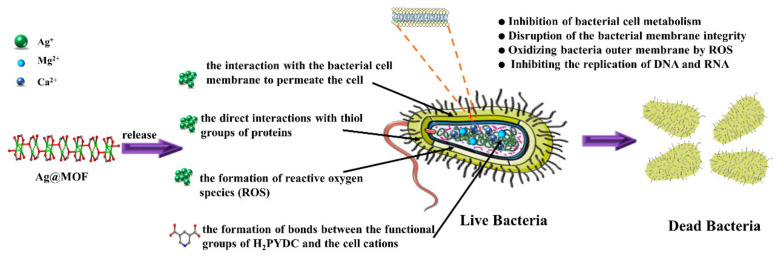
Antibacterial mechanisms of Ag-Metal-Organic Framework (Ag@MOF).

With this backdrop, we use PVA/Ag@MOF hydrogel as the inner layer and PVA/CS hydrogel as the outer layer to avoid direct contact between Ag@MOF and cells. The novelty of the present study is to evaluate the antibacterial activity of PVA hydrogels with the use of Ag@MOF for the first time in Figure 1. In addition, the physicochemical properties such as surface morphology, water retention properties, degradation properties, antibacterial activity, cytotoxicity and cell adhesion were also investigated in detail. Traditional tissue engineered scaffolds containing nano-silver hydrogels cannot effectively maintain their biocompatibility at low cost. The outer layer of the double-layer dressing has good biocompatibility, while the inner layer has high antibacterial activity and avoids direct contact with cells. Therefore, compared with traditional hydrogel tissue engineering scaffolds, the double-layer scaffolds are perfectly compatible with high antibacterial activity and good biocompatibility.

## 2. Materials and Methods

### 2.1. Materials

Polyvinyl alcohol (PVA: with an average degree of polymerization of 1799) and chitosan were provided by Aladdin reagent Shanghai Co., Ltd. (Shanghai, China). Pyridine-3, 5-dicarboxylic acid (H_2_PYDC) was purchased from Rhawn reagent Longxi Co., Ltd. AgNO_3_ was supplied by Sinopharm Chemical Reagent Co., Ltd. (St. Louis, MO, USA). All the chemicals were used as received without any further treatment. The deionized water was prepared by Millipore Milli-Q 18MΩ.

### 2.2. Synthesis of Ag@MOF

Ag@MOF [Ag_5_(PYDC)_2_(OH)] was synthesized under the modified hydrothermal according to our previous work [35]. Briefly, 0.14 g of H_2_PYDC was dispersed into 20 mL of deionized water to give the suspension and 0.32 g of AgNO_3_ was dissolved in suspension A to give suspension B. Suspension B was sonicated for 20 min and was sealed in a 50 mL Teflon-lined stainless steel autoclave and heated at 120 °C for 24 h. After cooling to room temperature, the colorless crystals were obtained through repeated centrifugation and redispersion with DI water (St. Louis, MO, USA). The Ag@MOF was dried at 80 °C for 12 h. The antibacterial mechanisms of Ag@MOF can be seen in Figure 1.

### 2.3. Preparation of Composites Hydrogels

#### 2.3.1. PVA Hydrogels

Briefly, 15 g of PVA was dissolved in 85 mL of DI water and stirred at 90 °C for 3 h till a homogeneous solution was obtained. The solution was frozen at 25 °C for 24 h and thawed at −25 °C for 8 h for three cycles before being washed with deionized water.

#### 2.3.2. PVA/CS Hydrogels

For the preparation of PVA/CS hydrogels, 2 g of chitosan was dissolved in 100 mL 1.5% (*v*/*v*) acetic acid solution, stirred at room temperature for 1 h to prepare chitosan solution. Then, 15 g of PVA was dissolved in 85 mL of DI water by stirring at 90 °C for 3 h. Mixing the two solutions and stirring at 80 °C for 1 h. The stirring speed at preparation of PVA/CS hydrogels was 200 rpm. Finally, the resulting solution was frozen at 25 °C for 24 h and thawed at −25°C for 8 h for three cycles before being washed with deionized water.

#### 2.3.3. PVA/Ag@MOF Hydrogels

PVA/Ag@MOF hydrogels was synthesized in PVA as a medium. 0.025 g Ag@MOF (0.5 wt%) was dissolved in 85 mL of DI water and sonicated for 30 min till a suspension was acquired. The mixture of 15 g PVA was dissolved in 85 mL of suspension by heating at 90 °C for 3 h. Finally, the resulting solution was frozen at 25 °C for 24 h and thawed at −25 °C for 8 h for three cycles before being washed with deionized water. The concentration of Ag@MOF was varied from 0.1 to 1 wt% and three hydrogels (0.1 wt%, 0.5 wt%, and 1 wt%) were prepared by same method.

#### 2.3.4. Bilayer Hydrogels

The prepared PVA/Ag@MOF cylindrical hydrogel is placed in a larger cylindrical container and then the PVA/CS solution is poured in to coat the PVA/Ag@MOF hydrogel. Finally, the resulting solution was frozen at 25 °C for 24 h and thawed at −25 °C for 8 h for three cycles before being washed with deionized water.

### 2.4. Characterization

#### 2.4.1. FT-IR

FT-IR spectra of the samples was recorded by a IRAffinity-1 spectrometer (Tokyo, Japan) attached to universal ATR accessory. The samples were analyzed from 600^−1^ to 4000 cm^−1^ with a scan rate of 2 cm^−1^.

#### 2.4.2. XRD

The XRD patterns of samples was recorded on X-Ray diffractometer (ULTIMALV, Japan Science Corporation, Tokyo, Japan) using Cu Kα radiation (40 kV, 40 mA, 1°/min from 5° to 80°, 0.05° scan amplitude).

#### 2.4.3. SEM

The morphology of samples was investigated using a JSM-6700 F scanning electron microscope (SEM) (JEOL, Japan). Ag@MOF nanoparticle was analyzed directly whereas composite hydrogels were analyzed by cross-section. The operating voltage was 5.00 kV.

#### 2.4.4. TGA

The thermal properties of the samples were recorded on SDT-Q600 thermal analyzer (St. Louis, MO, USA). The samples were heated from 25 °C to 600 °C at the rate of 10 °C/min under the flow of nitrogen.

#### 2.4.5. Rheological Characterization

The rheological measurements were measured by an MCR 101 rheometer (Anton Paar, Austria), which was fitted with a parallel plate geometry (diameter of 50 mm, a gap value of 1.2 mm). Dynamic viscoelastic measurement was performed in a constant strain (0.01%) to ensure that the deformation applied to the hydrogel was in a linear viscoelastic region. Viscoelastic properties of the lower layer were tested within the frequency range of 0.1–10 Hz.

#### 2.4.6. Water Retention Studies

The hydrogel was soaked in distilled water at 37 °C, and the equilibrium swelled hydrogel was placed in an oven at 37 °C. The hydrogel was weighed at predetermined intervals. The water retention (WR) for the hydrogel was calculated as follows:

(1) $$WR(\%)=\frac{W_{t}}{W_{e}}\times 100$$

where W_t_ is the weight of the hydrogel at time t and W_e_ is the swelling weight at equilibrium.

#### 2.4.7. In Vitro Degradation

In vitro biodegradation of wound dressings was tested by calculating the rate of weight loss in the presence of hydrolases encountered by the dressings at the site of injury [17]. The sample (2 × 1 cm^2^) was placed in phosphate-buffered saline (PBS) containing 10,000 U/mL lysozyme for enzymatic degradation. All samples were incubated at 37 °C for 25 days and the percentage of weightlessness was assessed at a pre-selected time point. The samples were removed and then freeze-dried at pre-selected time points. The percentage of weight loss was calculated as follows:

(2) $$\mathrm{Weight}\;\mathrm{loss}\left(\%\right)\;=\;[(W_{d}-W_{t})/W_{d}]\times \;100$$

where W_d_ is the weight of the sample before degradation and W_t_ is the weight of the sample after degradation.

#### 2.4.8. Antibacterial Properties

The antibacterial activity of the samples was measured against *E. coli* (ATCC25922) and *S. aureus* (ATCC6538) by agar disk diffusion test. The sterilized Petri dishes with LB broth and dissolved agar was heated for 15 min at 121 °C. The strains were inoculated into the Petri dishes at 37 °C for 24 h. The bactericidal effect of the samples was examined on culture dish inoculated with microbes. The strain was put on the agar plate, and spread evenly over the surface by sterile glass spreading rod. The samples were cut 5 mm discs and then placed on the agar plates. These plates were put in an incubator at 37 °C overnight. The diameters of inhibition zones were measured using a vernier calipers. All tests were performed in duplicate.

#### 2.4.9. Cytotoxicity Test

A549 cells were grown at 37 °C and 5% CO_2_ for 24 h in Dulbecco’ s modified Eagle medium supplemented with 10% fetal bovine serum and were seeded in 96-well plates at the density of 10^5^ cells and in a volume of 100 μL per well. Then, 20 μL of these polymer dispersions was added to the concentrations of 10, 20, and 50 μg/mL. The cells were incubation for 8, 16, and 24 h. After incubating for 8, 16, and 24 h, add 10μL CCK-8 reagent to per well, and then incubate for 1 h. The optical density was measured at 450 nm by a microplate reader (BioTek, EXL808, Qingdao, China).

#### 2.4.10. Cell Observations

After 5 days of culturing MDA-MB-231 cells and A549 cells on pure PVA hydrogels and PVA/CS hydrogels, the cells were gently rinsed with PBS (37 °C, pH 7.4) to remove non-adherent cells. Each sample was then fixed with 4% paraformaldehyde in PBS for 30 min, followed by permeabilization with 0.1% (*v*/*v*) Triton X-100 for 10 min. Afterwards, all samples were thoroughly rinsed 3 times with PBS and then observed under a fluorescent microscope (Olympus, Tokyo, Japan).

#### 2.4.11. Statistical Analysis

All experiments were carried out 3 times (*n* = 3), and the data are expressed as mean ± standard deviation. The results were analyzed with one-way ANOVA by SPSS (version 22.0, SPSS Inc., Chicago, IL, USA).

## 3. Results and Discussion

### 3.1. Structure and Morphology of Ag@MOF

**Figure 2:**
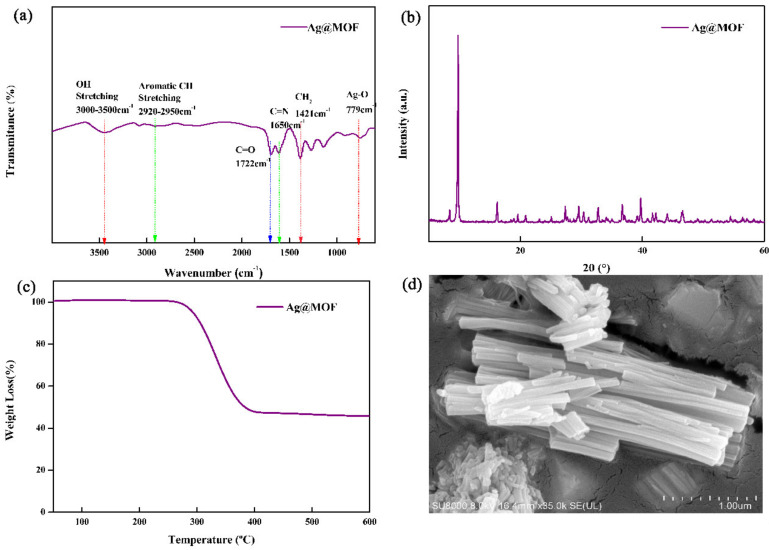
(**a**) FTIR spectra, (**b**) XRD pattern, (**c**) TGA curve and (**d**) SEM image of Ag@MOF.

To reveal structural changes of samples, XRD, FTIR, TGA and SEM of Ag@MOF have been analyzed. Figure 2a shows the FTIR spectra of Ag@MOF.

From the FTIR spectrum of Ag@MOF, the presence of broad peak at 3451 cm^−1^ corresponds to the vibrational stretching of O–H groups while peak at 2915 cm^−1^ is assigned to C-H stretching. The peaks at 1650 and 1722 cm^−1^ are attributed to C = N and C = O stretching vibration for the pyridine ring group, respectively. The peak near 779 cm^−1^ is assigned to Ag–O stretching vibrations, as previously reported [36]. This indicates that Ag^+^ is coordinated to the –COOH group in the H_2_PYDC ligand rather than to the -NH group [36].

The XRD pattern of Ag@MOF was shown in Figure 2b. On account of Ag@MOF, the diffraction peaks at 6.9°, 12.5°, and 15.6° are attributed to the (100), (110), and (112) crystal plane in the Ag@MOF, as reported before [35].

Figure 2c shows the TGA curves of Ag@MOF. The TGA curves of Ag@MOF reflects two step degradation: firstly, water is evaporated at 260 °C and secondly, the decomposition starts at 300 °C and accomplishes at 400 °C for hydroxyl, carboxylic, and pyridyl rings and mass loss is about 50%. After the temperature reached 400°C, Ag@MOF did not degrade further [35].

To view the morphology of samples, Ag@MOF is shown by SEM. The SEM image of Ag@MOF (Figure 2d) shows the rod-like structures, having the diameter of 0.3–1.2 μm and length of 3.0–9.0 μm. The results are consistent with previous reports [35].

### 3.2. SEM of the Hydrogels

**Figure 3:**
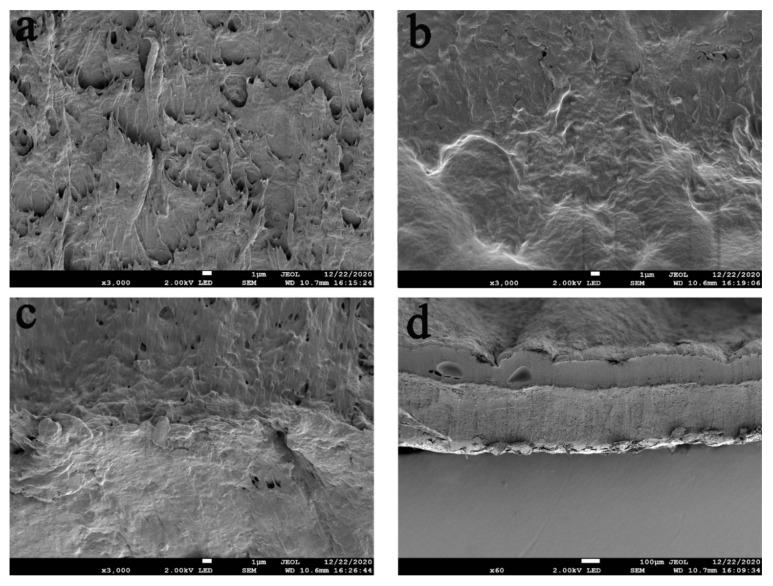
SEM images of (**a**) PVA, (**b**) PVA/CS (outer hydrogel), (**c**) PVA/Ag@MOF (inner hydrogel), and (**d**) Bilayer hydrogel.

The morphology of the hydrogel was analyzed using scanning electron microscopy [10]. Figure 3 shows the cross-sectional images of the hydrogels. The hydrogel has a three-dimensional network structure as can be seen from the figures. There are interconnected micropores in the hydrogel structure which are evenly distributed and allow the penetration of oxygen and nutrients, thus the hydrogel can be regarded as an ECM [2]. In addition, a clear bilayer structure can be seen in Figure 3d, indicating that the bilayer hydrogel scaffold was successfully prepared. In addition, the picture of PVA is in Figure S2 of Supplementary Materials.

### 3.3. Rheological Analysis of Hydrogels

**Figure 4:**
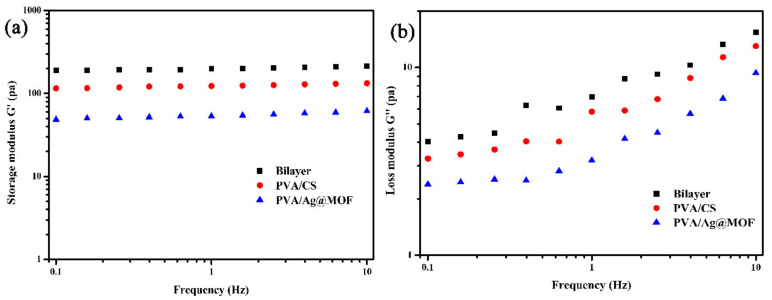
Frequency of (**a**) storage modulus (G′) and (**b**) loss modulus (G″) of PVA/CS (outer hydrogel), PVA/Ag@MOF (inner hydrogel), and Bilayer hydrogel.

Figure 4 exhibits typical gel rheological behaviour by the linear viscoelastic frequency scan response. The storage modulus (G’) of the hydrogels is much higher than the loss modulus (G″). PVA/Ag@MOF, bilayer, and PVA/CS show rheological properties in ascending order. This indicates that the hydrogels are highly elastic [37]. This suggests that the addition of Ag@MOF has a negative effect on the hydrogen bonding crosslinking of PVA and reduces the density of the polymer network. The rheological properties of the bilayer hydrogel, on the other hand, are much higher than those of commercial scaffold.

### 3.4. Water Retention of Hydrogels

**Figure 5:**
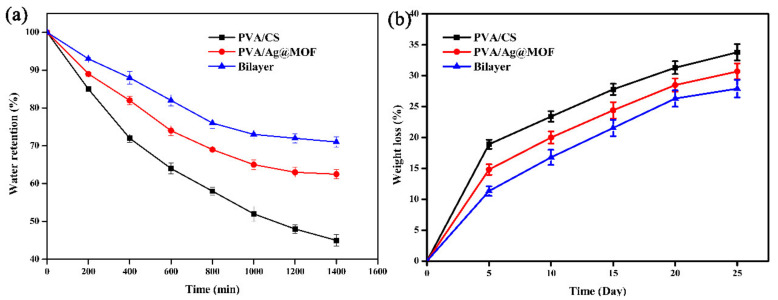
(**a**) Water retention and (**b**) degradation of PVA/CS (outer hydrogel), PVA/Ag@MOF (inner hydrogel), and Bilayer hydrogel.

Figure 5a exhibits the results of water retention. PVA/CS, PVA/Ag@MOF, and bilayer showed water retention in ascending order. This may be due to the rigid hydrophobic structure of Ag@MOF and the layered structure of bilayer hydrogels [36]. The higher water retention of the hydrogels can prevent the hydrogels from dissolving or deforming and causing negative effects on the body. In addition, hydrogels has a porous spatial structure, which is conducive to gaseous exchange and provides a site for cell adhesion and proliferation.

### 3.5. Biodegradation of Hydrogels

The degradation rate of the tissue engineered scaffold should match the regeneration rate of the damaged tissue to ensure effective cell proliferation and growth [5]. Figure 5 shows the degradation curves of PVA/CS, PVA/Ag@MOF, bilayer hydrogels. PVA/CS, PVA/Ag@MOF, and bilayer show degradation rate in descending order. This may be because the addition of Ag@MOF leads to the increase in crystallinity and decrease in hydrophilicity of nanocomposites, which reduces the number of enzyme molecules containing water diffusing into the mesh, thus reducing the degradation rate [38]. However, the low crystallinity of PVA/CS leads to a large exposure of the macromolecular chains to invading enzyme molecules, resulting in a large degradation of the PVA/CS hydrogel.

### 3.6. Antibacterial Properties of Hydrogels

**Figure 6:**
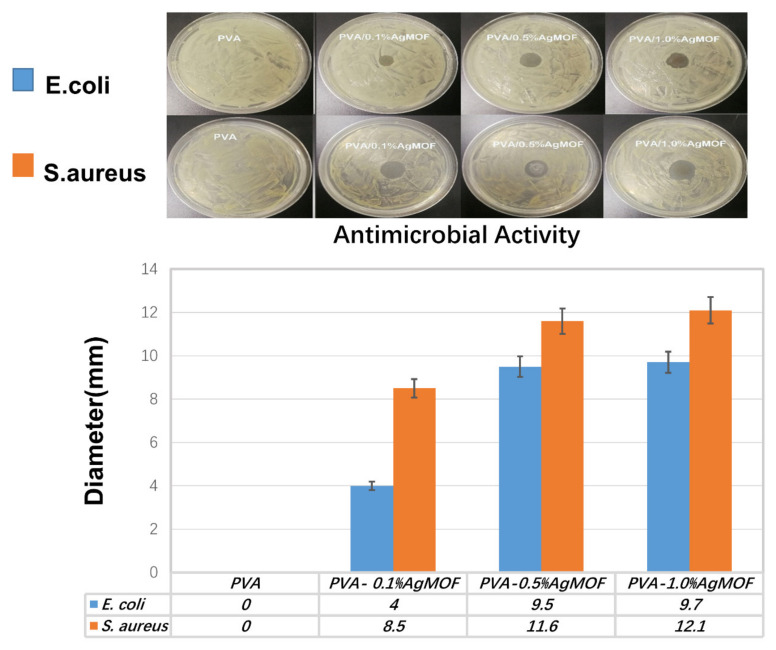
Images and diameters of inhibition zones for PVA and PVA/Ag@MOF hydrogels against *E. coli* and *S. aureus*.

Tissue engineering scaffold should have good antibacterial activity to prevent a serious risk of infection [21]. The antimicrobial activity of composites hydrogel was tested against *S. aureus* and *E. coli* which represent Gram-positive and Gram-negative bacteria, respectively in Figure 6. The diameter of inhibition zone is related to the sensitivity of the hydrogels to microorganisms in the disk diffusion test. The PVA have hardly any antibacterial activity. PVA/Ag@MOF hydrogels exhibited excellent antibacterial activity on both Gram-negative and Gram-positive bacteria. PVA, PVA/0.1%Ag@MOF, PVA/0.5%Ag@MOF, and PVA/1.0%Ag@MOF show antimicrobial activity in ascending order. Due to its relatively large size, Ag@MOF cannot penetrate bacterial cell membranes. PVA/Ag@MOF can release Ag^+^ to strongly attract the enzyme protein in bacteria, and quickly bind together to destroy the bacterial cell membrane. Ag^+^ can also form reactive oxygen species (ROS) that further attack cell membranes [35]. In addition, Ag@MOF can cause the functional groups of organic ligand to form bonds with the cations of cells, further promoting bacterial death.

### 3.7. Biocompatibility and Cell Adhesion of Hydrogels

**Figure 7:**
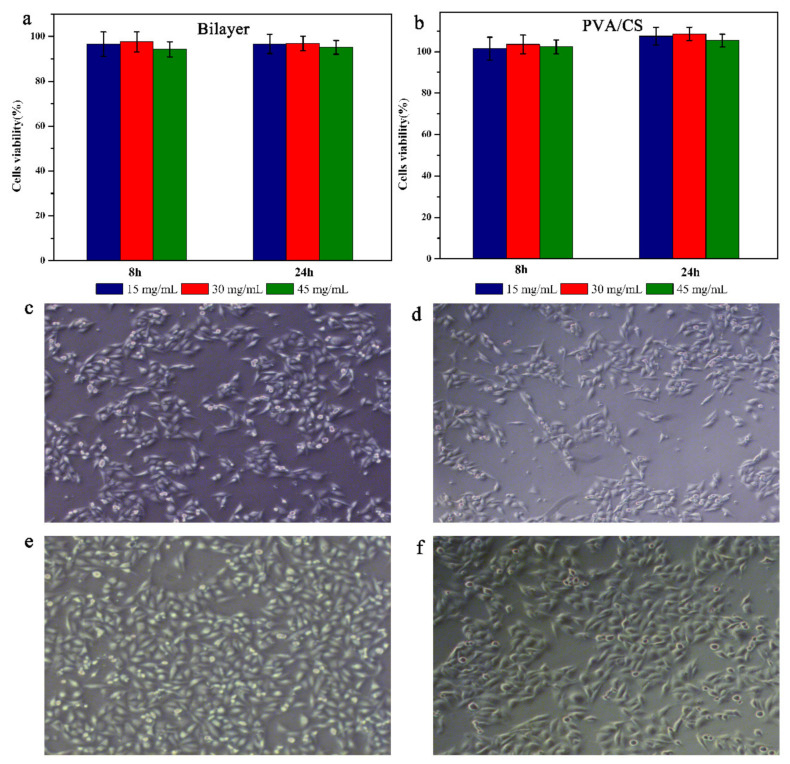
Viability of A549 cells treated with increasing concentrations of (**a**) PVA/CS and (**b**) bilayer hydrogels for 8, 16, and 24 h; Optical microscope images (100×) of (**c**,**d**) MDA-MB-231 cells and (**e**,**f**) A549 cells cultured on the surface of PVA/CS and bilayer hydrogels.

Good biocompatibility is an important factor in tissue engineered scaffold [39]. In order to further evaluate the cytocompatibility of these samples, the samples were tested for cytotoxicity. Figure 7a shows the viability of A549 cells after 8 and 24 h of incubation. As can be seen in Figure 7b, the cell viability of all samples was above 90%, indicating that the bilayer hydrogel was non-toxic to A549 cells. The good cell viability of the hydrogels is mainly due to the bilayer structure which avoids direct contact between Ag@MOF and cells, and the green preparation process of the hydrogel. In this case, the release of Ag^+^ may be a fairly small number and balanced with molecules that are beneficial to the cells, such as carbon, which is a source of energy and allows the cell to proliferate.

MDA-MB-231 cells and A549 cells were cultured on approximately 3 mm thick hydrogel films to observe their adhesion on bilayer hydrogels and PVA/CS hydrogels. After 5 d of culture, A549 cells cultured on both the bilayer hydrogel and PVA/CS hydrogel were spindle-shaped, indicating good cell adhesion and spreading. MDA-MB-231 cells also showed a similar phenomenon. These results suggest that the bilayer hydrogels have better cell adhesion properties, which is related to the slow release of Ag^+^ from the inner PVA/Ag@MOF layer to the outer surface. the slow release of Ag^+^ increases the roughness of the outer surface and improves the cell adhesion ability of the bilayer hydrogels.

## 4. Conclusions

In this study, the bilayer composite hydrogels composed of PVA/Ag@MOF and PVA/CS as inner and outer layers, respectively, were successfully prepared for tissue engineering. The excellent pore size distribution, water retention, biocompatibility and cell adhesion of the outer layer (PVA/CS) make it suitable for tissue engineering scaffolds. The good antibacterial activity of the inner PVA/Ag@MOF can prevent bacterial infection. PVA, PVA/0.1%Ag@MOF, PVA/0.5%Ag@MOF, and PVA/1.0%Ag@MOF show antimi-crobial activity in ascending order. The bilayer hydrogel scaffold combines the advantages of the inner and outer layers. The cell viability of all samples was above 90%, indicating that the bilayer hydrogel was non-toxic to A549 cells. Therefore, new bilayer composite proved to be a promising application that can be used as lung tissue engineering scaffold.

## Data Availability

Not applicable.

## Supplementary Materials

The following are available online at https://www.mdpi.com/article/10.3390/polym13183151/s1, Figure S1: Cumulative release profiles of Ag, Figure S2: digital image of bilayer.

## References

[1] Guzelgulgen M., Ozkendir-Inanc D., Yildiz U.H., Arslan-Yildiz A. (2021). Glucuronoxylan-based quince seed hydrogel: A promising scaffold for tissue engineering applications. Int. J. Biol. Macromol..

[2] Jiang Z., Zhang K., Du L., Cheng Z., Zhang T., Ding J., Li W., Xu B., Zhu M. (2021). Construction of chitosan scaffolds with controllable microchannel for tissue engineering and regenerative medicine. Mater. Sci. Eng. C.

[3] Laird N.Z., Acri T.M., Chakka J.L., Quarterman J.C., Malkawi W.I., Elangovan S., Salem A.K. (2021). Applications of nanotechnology in 3D printed tissue engineering scaffolds. Eur. J. Pharm. Biopharm..

[4] Laird N.Z., Acri T.M., Tingle K., Salem A.K. (2021). Gene- and RNAi-activated scaffolds for bone tissue engineering: Current progress and future directions. Adv. Drug Deliv. Rev..

[5] Ma P., Wu W., Wei Y., Ren L., Lin S., Wu J. (2021). Biomimetic gelatin/chitosan/polyvinyl alcohol/nano-hydroxyapatite scaffolds for bone tissue engineering. Mater. Des..

[6] Poormoghadam D., Ghollasi M., Babavalian H., Tabasi A., Shams M., Goodarzi V., Salimi A. (2021). Modification and characterization of an innovative polyvinyl alcohol-45S5 bioactive glass nanocomposite scaffold containing Donepezil hydrochloride for bone tissue engineering applications. Mater. Lett..

[7] Sani I.S., Rezaei M., Khoshfetrat A.B., Razzaghi D. (2021). Preparation and characterization of polycaprolactone/chitosan-g-polycaprolactone/hydroxyapatite electrospun nanocomposite scaffolds for bone tissue engineering. Int. J. Biol. Macromol..

[8] Xu J., Fang H., Zheng S., Li L., Jiao Z., Wang H., Nie Y., Liu T., Song K. (2021). A biological functional hybrid scaffold based on decellularized extracellular matrix/gelatin/chitosan with high biocompatibility and antibacterial activity for skin tissue engineering. Int. J. Biol. Macromol..

[9] Zuluaga-Vélez A., Quintero-Martinez A., Orozco L.M., Sepúlveda-Arias J.C. (2021). Silk fibroin nanocomposites as tissue engineering scaffolds—A systematic review. Biomed. Pharmacother..

[10] Ravishankar K., Venkatesan M., Desingh R.P., Mahalingam A., Sadhasivam B., Subramaniyam R., Dhamodharan R. (2019). Biocompatible hydrogels of chitosan-alkali lignin for potential wound healing applications. Mater. Sci. Eng. C.

[11] Khan M.I., Paul P., Behera S.K., Jena B., Tripathy S.K., Stålsby Lundborg C., Mishra A. (2020). To decipher the antibacterial mechanism and promotion of wound healing activity by hydrogels embedded with biogenic Ag@ZnO core-shell nanocomposites. Chem. Eng. J..

[12] Akhlaq M., Azad A., Ullah I., Nawaz A., Safdar M., Bhattacharya T., Uddin A., Abbas S., Mathews A., Kundu S. (2021). Methotrexate-Loaded Gelatin and Polyvinyl Alcohol (Gel/PVA) Hydrogel as a pH-Sensitive Matrix. Polymers.

[13] Figueroa-Pizano M., Vélaz I., Peñas F., Zavala-Rivera P., Rosas-Durazo A., Maldonado-Arce A., Martínez-Barbosa M. (2018). Effect of freeze-thawing conditions for preparation of chitosan-poly (vinyl alcohol) hydrogels and drug release studies. Carbohydr. Polym..

[14] Lee S.J., Nah H., Heo D.N., Kim K.-H., Seok J.M., Heo M., Moon H.-J., Lee D., Lee J.S., An S.Y. (2020). Induction of osteogenic differentiation in a rat calvarial bone defect model using an In situ forming graphene oxide incorporated glycol chitosan/oxidized hyaluronic acid injectable hydrogel. Carbon.

[15] Liang Y., Zhao X., Hu T., Chen B., Yin Z., Ma P.X., Guo B. (2019). Adhesive Hemostatic Conducting Injectable Composite Hydrogels with Sustained Drug Release and Photothermal Antibacterial Activity to Promote Full-Thickness Skin Regeneration During Wound Healing. Small.

[16] Aradmehr A., Javanbakht V. (2020). A novel biofilm based on lignocellulosic compounds and chitosan modified with silver nanoparticles with multifunctional properties: Synthesis and characterization. Colloids Surfaces A: Physicochem. Eng. Asp..

[17] Biranje S.S., Madiwale P.V., Patankar K.C., Chhabra R., Bangde P., Dandekar P., Adivarekar R.V. (2020). Cytotoxicity and hemostatic activity of chitosan/carrageenan composite wound healing dressing for traumatic hemorrhage. Carbohydr. Polym..

[18] Cazón P., Vázquez M., Velazquez G. (2018). Composite films of regenerate cellulose with chitosan and polyvinyl alcohol: Evaluation of water adsorption, mechanical and optical properties. Int. J. Biol. Macromol..

[19] Bakhsheshi-Rad H.R., Ismail A.F., Aziz M., Akbari M., Hadisi Z., Omidi M., Chen X. (2020). Development of the PVA/CS nanofibers containing silk protein sericin as a wound dressing: In vitro and in vivo assessment. Int. J. Biol. Macromol..

[20] Lee S., Lei Y., Wang D., Li C., Cheng J., Wang J., Meng W., Liu M. (2019). The Study of Zeolitic Imidazolate Framework (ZIF-8) Doped Polyvinyl Alcohol/Starch/Methyl Cellulose Blend Film. Polymers.

[21] Niua X., Weiab Y., Liuc Q., Yangc B., Mac N., Lic Z., Zhaoab L., Chenab W., Huangab D. (2020). Silver-loaded microspheres reinforced chitosan scaffolds for skin tissue engineering. Eur. Polym. J..

[22] Kovacova M., Markovic Z.M., Humpolíček P., Mičušík M., Švajdlenková H., Kleinová A., Danko M., Kubát P., Vajďák J., Capáková Z. (2018). Carbon Quantum Dots Modified Polyurethane Nanocomposite as Effective Photocatalytic and Antibacterial Agents. ACS Biomater. Sci. Eng..

[23] Slenters T.V., Hauser-Gerspach I., Daniels A.U., Fromm K.M. (2008). Silver coordination compounds as light-stable, nano-structured and anti-bacterial coatings for dental implant and restorative materials. J. Mater. Chem..

[24] Huang F., Gao Y., Zhang Y., Cheng T., Ou H., Yang L., Liu J., Shi L., Liu J. (2017). Silver-Decorated Polymeric Micelles Combined with Curcumin for Enhanced Antibacterial Activity. ACS Appl. Mater. Interfaces.

[25] Patel K.K., Surekha D.B., Tripathi M., Anjum M., Muthu M.S., Tilak R., Agrawal A.K., Singh S. (2019). Antibiofilm Potential of Silver Sulfadiazine-Loaded Nanoparticle Formulations: A Study on the Effect of DNase-I on Microbial Biofilm and Wound Healing Activity. Mol. Pharm..

[26] Bateman F.L., Kirejczyk S.G.M., Stewart G.V., Cutler D.C., Quilling L.L., Howerth E.W., Mayer J. (2019). Effects of an enrofloxacin-silver sulfadiazine emulsion in the ears of rabbits with perforated tympanic membranes. Am. J. Veter- Res..

[27] Wang Z., Wang T., Hua A., Ma S., Zhang Z., Liu L. (2019). Prolonged antimicrobial activity of silver core-carbon shell nanoparticles. Korean J. Chem. Eng..

[28] Tian X., Jiang X., Welch C., Croley T.R., Wong T.-Y., Chen C., Fan S., Chong Y., Li R., Ge C. (2018). Bactericidal Effects of Silver Nanoparticles on Lactobacilli and the Underlying Mechanism. ACS Appl. Mater. Interfaces.

[29] Travlou N.A., Algarra M., Alcoholado C., Cifuentes-Rueda M., Labella A.M., Lázaro-Martínez J.M., Rodriguez-Castellon E., Bandosz T.J. (2018). Carbon Quantum Dot Surface-Chemistry-Dependent Ag Release Governs the High Antibacterial Activity of Ag-Metal–Organic Framework Composites. ACS Appl. Bio Mater..

[30] He Y., Zhou W., Qian G., Chen B. (2014). Methane storage in metal–organic frameworks. Chem. Soc. Rev..

[31] Barea E., Montoro C., Navarro J. (2014). Toxic gas removal-metal–organic frameworks for the capture and degradation of toxic gases and vapours. Chem. Soc. Rev..

[32] Zhu Y., Zhang Z., Li W., Lei Z., Cheng N., Tan Y., Mu S., Sun X. (2019). Highly Exposed Active Sites of Defect-Enriched Derived MOFs for Enhanced Oxygen Reduction Reaction. ACS Sustain. Chem. Eng..

[33] Mukherjee S., Ganguly S., Chakraborty A., Mandal A., Das D. (2018). Green Synthesis of Self Assembled Nanospherical Dysprosium MOFs: Selective and Efficient Detection of Picric Acid in Aqueous and Gas Phase. ACS Sustain. Chem. Eng..

[34] Liu M., Wang L., Zheng X., Xie Z. (2017). Zirconium-Based Nanoscale Metal–Organic Framework/Poly(ε-caprolactone) Mixed-Matrix Membranes as Effective Antimicrobials. ACS Appl. Mater. Interfaces.

[35] Lu X., Ye J., Zhang D., Xie R., Bogale R.F., Sun Y., Zhao L., Zhao Q., Ning G. (2014). Silver carboxylate metal–organic frameworks with highly antibacterial activity and biocompatibility. J. Inorg. Biochem..

[36] Zhang M., Wang G., Wang D., Zheng Y., Li Y., Meng W., Zhang X., Du F., Lee S. (2021). Ag@MOF-loaded chitosan nanoparticle and polyvinyl alcohol/sodium alginate/chitosan bilayer dressing for wound healing applications. Int. J. Biol. Macromol..

[37] Qi X., Hu X., Wei W., Yu H., Li J., Zhang J., Dong W. (2015). Investigation of Salecan/poly(vinyl alcohol) hydrogels prepared by freeze/thaw method. Carbohydr. Polym..

[38] Bagri L.P., Saini R.K., Bajpai A.K., Choubey R. (2019). Silver hydroxyapatite reinforced poly(vinyl alcohol)—starch cryogel nanocomposites and study of biodegradation, compressive strength and antibacterial activity. Polym. Eng. Sci..

[39] Zandi N., Dolatyar B., Lotfi R., Shallageh Y., Shokrgozar M.A., Tamjid E., Annabi N., Simchi A. (2021). Biomimetic nanoengineered scaffold for enhanced full-thickness cutaneous wound healing. Acta Biomater..

