# Supplementary material for: Polyvinyl Alcohol/Chitosan and Polyvinyl Alcohol/Ag@MOF Bilayer Hydrogel for Tissue Engineering Applications
Source: Polymers (Basel). 2021 Sep 17;13(18):3151. doi: 10.3390/polym13183151 (PMC8468989; doi:10.3390/polym13183151)
Supplement: Supplementary file 1 [file polymers-13-03151-s001.zip › polymers-1358646-supplementary.pdf]

Polyvinyl alcohol/chitosan and polyvinyl alcohol/Ag@MOF bi-layer hydrogel for tissue engineering applications

3. Results and Discussion

3.1. Ag Ion Release of the Bilayer

The results shown in Figure S1 demonstrate that PVA/Ag@MOF possessed the maximum amount of release. In addition, the release amount of bilayer and PVA/Ag@MOF was 17.24 and 45.13 μg/cm2, respectively, at 24 h. The cytotoxicity is marginal when the amount of release is small.

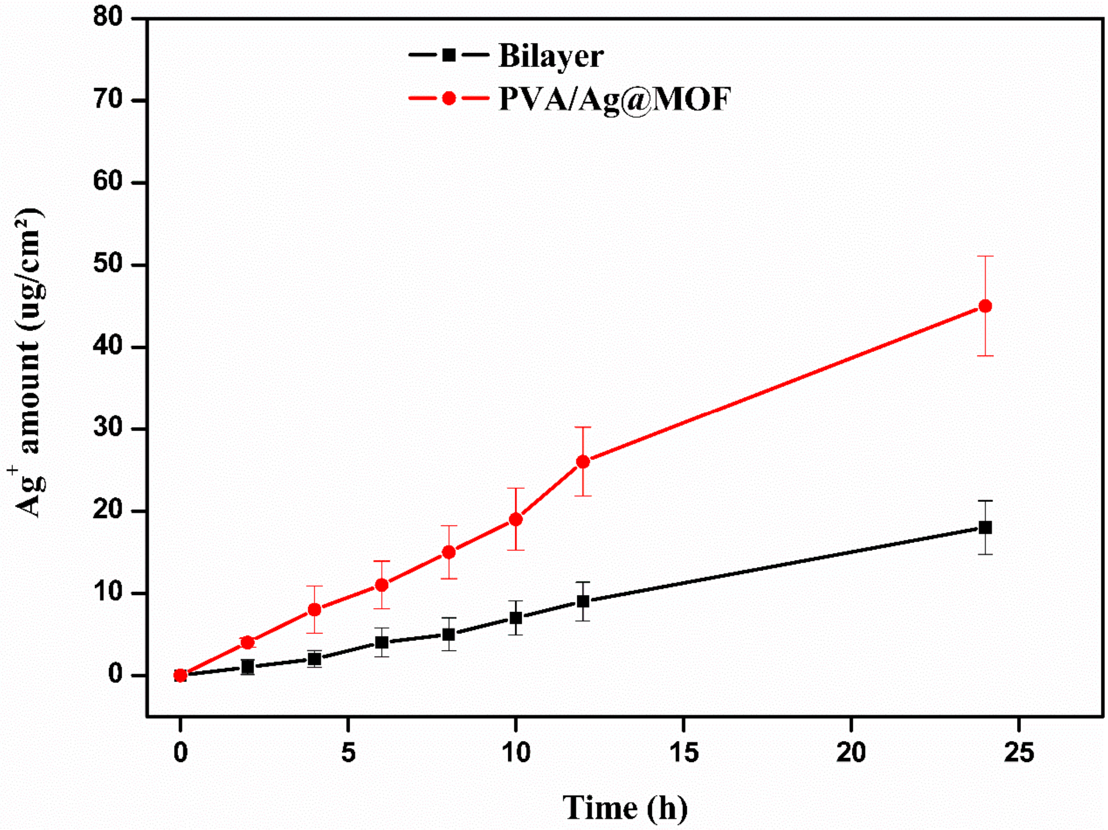

Figure S1. Cumulative release profiles of Ag.

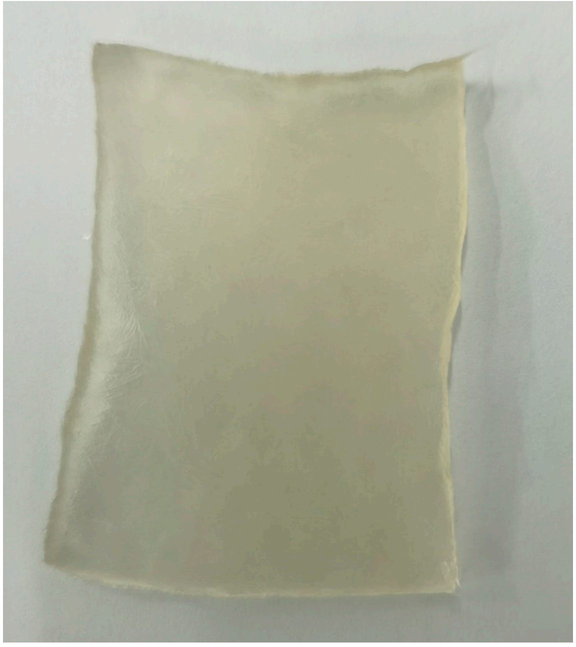

Figure S2. digital image of bilayer.
